# An Alternating Current Dental Engine

**Published:** 1900-10-15

**Authors:** 


### AN ALTERNATING CURRENT DENTAL ENGINE.

In no branch of electrical work have the improvements of recent years been more marked than in the manufacture of electrical appliances for dental use.

There is, however, one problem with which manufacturers have been unsuccessfully struggling for a great many years, and that is to produce a simple and reliable electric motor for dental use which could be attached directly to the alternating current lighting systems now so frequently found in many of our cities, and especially in the country towns.

The 110-volt direct current motor has probably been developed to the highest state of the art, so also has the storage battery outfit which is very reliable, and is being used extensively by those dentists who have no day supply of electricity.

But the absence until now of a satisfactory dental motor for the alternating current has been a great obstacle to those dentists whose source of supply is from the alternating systems, and the appearance of a reliable motor is thus correspondingly welcome.

As was to be expected, in the wild rush for development in the larger class of alternating current appliances, the small alternating dental motor was long delayed, which accounts perhaps for the fact that when its advent was finally made its success was instantaneous.

It is with pleasure that we refer to the beautiful little motor recently brought to our attention by The Ritter Dental Mfg. Co., of Rochester, N. Y.

Its construction presents considerable ingenuity in design, and much care has been expended on its many novel details.

Those who contemplate purchasing one of these engines must inquire of the electric light company who furnish them the electricity the voltage of the current, and the number of alternations per minute. It is necessary for the manufacturers to know this before they can supply a motor suited to the current.

The speed regulations are just as good as those of the direct current motor, and the manufacturers state that this can be varied from about 1,000 revolutions per minute, the slowest, to 3,600 revolutions per minute, and the power is practically the same throughout this range.

A single lever on the controller starts the motor, changes the speed, instantly stops it, and reverses the direction of rotation of the bur.

The motor is noiseless in operation, and responds readily to the movements of the controller lever.

